# Primary breast diffuse large B-cell lymphoma mimicking breast carcinoma: a case report

**DOI:** 10.3389/fonc.2026.1911895

**Published:** 2026-08-17

**Authors:** Renda Jiang, Dalei Chen, Luning Wu, Liangying Zhao, Guinv Hu

**Affiliations:** Department of Breast Surgery, Dongyang Hospital Affiliated to Wenzhou Medical University, Dongyang, China

**Keywords:** breast mass, diffuse large B-cell lymphoma, extranodal lymphoma, primary breast lymphoma, R-CHOP

## Abstract

**Background:**

Primary breast lymphoma (PBL) is an exceedingly rare extranodal malignancy that frequently mimics breast carcinoma on clinical and radiological examination, posing a significant diagnostic challenge.

**Case presentation:**

We report a case of a 39-year-old Chinese woman who presented with two palpable right breast masses. Imaging revealed suspicious lesions classified as Breast Imaging-Reporting and Data System 4A, prompting surgical excision and biopsy. Histopathological examination and immunohistochemical (IHC) analysis confirmed a diagnosis of diffuse large B-cell lymphoma, germinal center B-cell subtype. Staging by 18F-fluorodeoxyglucose positron emission tomography/computed tomography established disease as Ann Arbor stage IE, asymptomatic (stage IEA) with an International Prognostic Index score of 0. The patient was initiated on R-CHOP chemoimmunotherapy and demonstrated a favorable response after three cycles, with no evidence of recurrence.

**Conclusion:**

This case highlights the importance of histopathological and IHC evaluation in differentiating PBL from breast carcinoma. Early and accurate diagnosis is essential to guide appropriate treatment and optimize patient outcomes.

## Introduction

Primary breast lymphoma (PBL) is a rare extranodal malignancy, accounting for approximately 0.04–0.5% of all breast malignancies and 1–2% of extranodal non-Hodgkin lymphomas (1, 2). Diffuse large B-cell lymphoma (DLBCL) represents the most common histological subtype of PBL. Owing to its nonspecific clinical manifestations and imaging features that closely resemble those of breast carcinoma, PBL is frequently misdiagnosed, often leading to inappropriate surgical management (3). Definitive diagnosis relies on histopathological examination and immunohistochemical (IHC) profiling. Here, we present a case of PBL-DLBCL in a 39-year-old woman initially suspected to have primary breast carcinoma, discuss the diagnostic approach, and review the relevant literature.

## Case presentation

### Clinical presentation

A 39-year-old Chinese woman presented with a right breast mass that she had first noticed on self-examination one week before presentation, without any identifiable precipitating cause. She denied breast pain, nipple discharge, skin changes, fever, night sweats, and weight loss. Her past history was notable for two cesarean sections performed within the past ten years; apart from these, she had no prior surgical history and denied any other comorbidities. There was no family history of breast carcinoma, lymphoma, or other malignancies, and no known hereditary or genetic disorders. Psychosocial history was unremarkable. Physical examination revealed two distinct palpable masses in the right breast: a mass approximately 1.0 cm in diameter in the upper outer quadrant, and a larger mass approximately 4.0 cm in diameter lateral to the right nipple. Both masses were firm in consistency, mobile, and had indistinct margins. Neither mass was tender; the overlying skin was normal, and no enlarged lymph nodes were detected in the bilateral axillae. The patient had an Eastern Cooperative Oncology Group (ECOG) performance status of 0. Laboratory investigations revealed normal serum levels of carcinoembryonic antigen (CEA) and cancer antigen 15-3 (CA15-3), a normal complete blood count, normal sex hormone levels, and normal coagulation function. Serum lactate dehydrogenase (LDH) was 160 U/L (within normal limits). Serum biochemistry showed mildly elevated bilirubin (total bilirubin 25.1 μmol/L, direct bilirubin 7.3 μmol/L, and indirect bilirubin 17.8 μmol/L), with all other biochemical parameters within normal limits. Immunological profiling showed mildly decreased lymphocyte subsets (lymphocytes 1410 × 10^6/L; CD3+ T lymphocytes 965.3 × 10^6/L; CD3+CD8+ suppressor T lymphocytes 336.0 × 10^6/L), with all other immunological parameters within normal limits.

## Imaging findings

**Figure 1:**
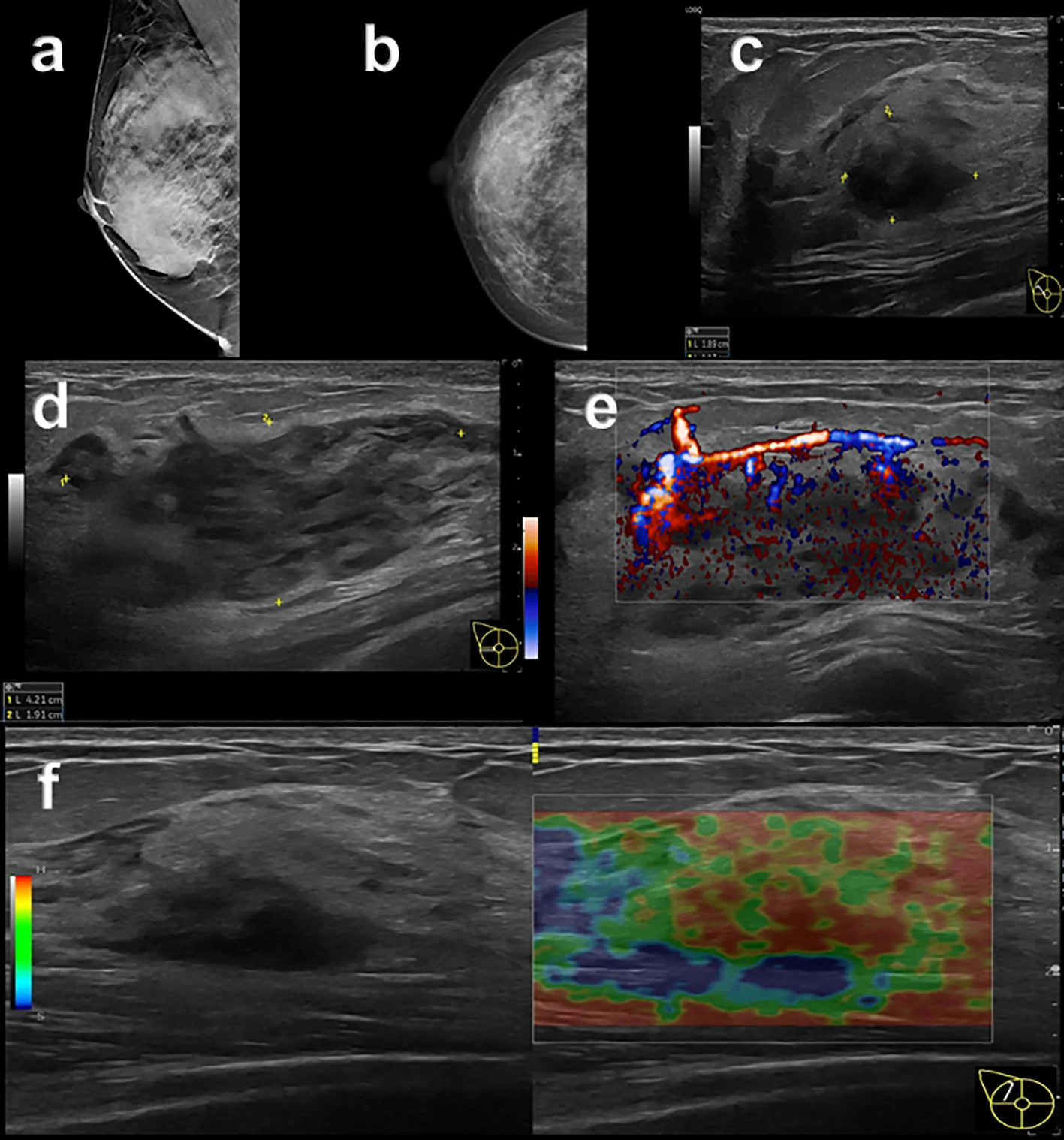
Preoperative imaging findings of the right breast. **(a)** Digital breast tomosynthesis (mediolateral oblique slice) and **(b)** full-field digital mammography (craniocaudal 2D view), showing a periareolar focal asymmetry with indistinct margins within a heterogeneously dense right breast (ACR category C). **(c)** Ultrasonography at the 10 o’clock position: an oval to lobulated mass with obscured margins and heterogeneous echogenicity. **(d)** Ultrasonography at the 9 o’clock position: a lobulated hypoechoic mass with indistinct margins and a peripheral echogenic rind. **(e)** Color Doppler flow imaging (CDFI) demonstrating predominantly peripheral vascularity in the 9 o’clock lesion. **(f)** Elastography showing intermediate-to-hard tissue stiffness of the 9 o’clock lesion (H, hard; S, soft). Both lesions were classified as BI-RADS category 4A.

Mammography demonstrated an oval-shaped focal asymmetry with indistinct margins in the periareolar region of the right breast, measuring approximately 4.0 cm in diameter, within a heterogeneously dense background (American College of Radiology [ACR] category C) (**Figures 1a, b**). Ultrasonography revealed an oval to lobulated mass with obscured margins and heterogeneous echogenicity at the 10 o’clock position of the right breast, measuring 19 × 12 × 14 mm. At the 9 o’clock position, an additional lobulated hypoechoic mass measuring approximately 42 × 19 mm demonstrated indistinct margins, demonstrated indistinct margins, a heterogeneous internal echo pattern with disorganized glandular architecture, multiple punctate hyperechoic foci, and cystic anechoic regions, as well as a peripheral echogenic rind. Both lesions demonstrated parallel orientation relative to the skin surface (**Figures 1c, d**). Color Doppler flow imaging (CDFI) demonstrated predominantly peripheral vascularity at the 9 o’clock position (**Figure 1e**). Elastography of the larger lesion revealed intermediate-to-hard tissue stiffness (**Figure 1f**). The largest ipsilateral axillary lymph node measured 14 × 6 mm with preserved morphology. Both lesions were classified as Breast Imaging-Reporting and Data System (BI-RADS) category 4A (4).

## Surgical and pathological findings

**Figure 2:**
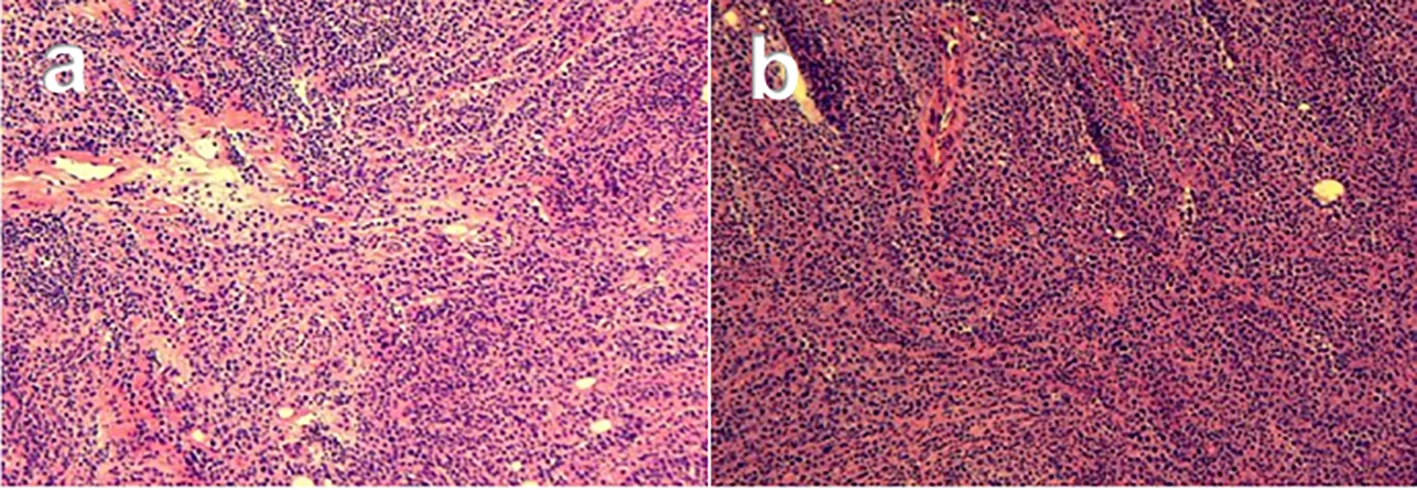
Intraoperative frozen section histopathology of the right breast masses (hematoxylin and eosin staining, ×40). **(a)** 9 o’clock lesion; **(b)** 10 o’clock lesion. Both frozen sections demonstrate atypical cellular proliferates suspicious for a lymphohematopoietic neoplasm.

Given the radiological suspicion for malignancy, the patient underwent surgical excision and biopsy. Two discrete lumps, one section from each lesion (9 o’clock and 10 o’clock positions), were submitted for intraoperative frozen section examination. Both masses were excised completely, together with a 1-cm margin of surrounding grossly normal breast tissue, to ensure complete removal of the lesions. On gross examination, the excised nodules were relatively well circumscribed, solid, and grayish-white on cut section, with a homogeneous, fish-flesh-like appearance. Intraoperative frozen section analysis identified atypical cells within the tumor tissue at both positions, raising the suspicion of a lymphohematopoietic neoplasm, while an epithelial origin could not be entirely excluded (**Figures 2a, b**).

**Figure 3:**
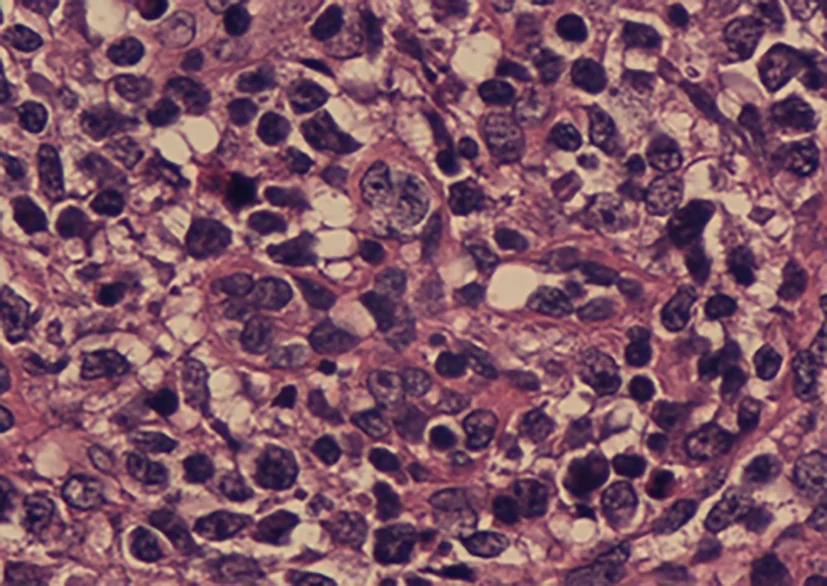
Permanent section histopathology of the right breast mass (hematoxylin and eosin staining, ×400). Diffuse proliferation of atypical lymphoid cells with abundant clear/vacuolated cytoplasm, relatively low N:C ratio, irregular nuclear contours, vesicular chromatin, and prominent nucleoli, arranged in cohesive nests and diffuse sheet-like patterns.

Definitive histopathological examination of the permanent sections demonstrated diffuse proliferation of atypical lymphoid cells with abundant clear cytoplasm, irregular nuclear contours, and prominent nucleoli, arranged in cohesive nests and diffuse sheet-like patterns (**Figure 3**). The final pathological diagnosis for both lesions was a lymphoproliferative neoplasm consistent with DLBCL, germinal center B-cell (GCB) subtype. Tumor dimensions were 4.5 × 3.5 × 2.5 cm at the 9 o’clock position and 3.5 × 2.5 × 2.0 cm at the 10 o’clock position.

## Immunohistochemical analysis

**Figure 4:**
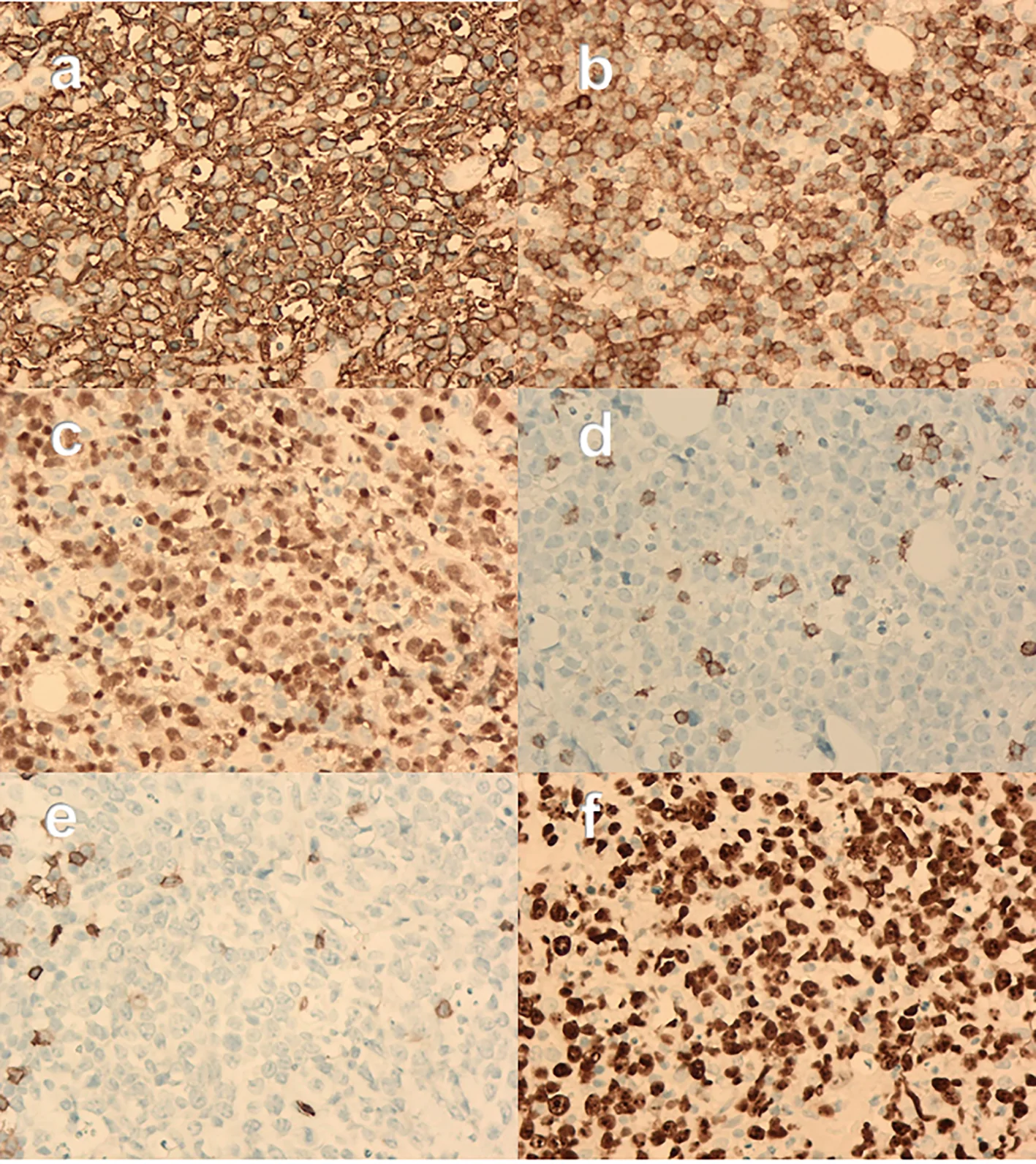
Immunohistochemical staining panel of the right breast mass. **(a)** CD20: diffuse membranous positivity; **(b)** CD79a: diffuse positivity; **(c)** PAX-5: diffuse nuclear positivity; **(d)** CD3: focally positive in background reactive T-lymphocytes; **(e)** CD5: focally positive in background reactive T-lymphocytes; **(f)** Ki-67: nuclear positivity in approximately 80% of tumor cells.

IHC profiling demonstrated diffuse positive expression of B-cell lineage markers including CD20 (**Figure 4a**), CD79a (**Figure 4b**), and PAX-5 (**Figure 4c**). T-cell markers CD3 (**Figure 4d**) and CD5 (**Figure 4e**) were also focally positive in background reactive T-lymphocytes infiltrating the tumor bed. The Ki-67 proliferation index was approximately 80%, indicating a high proliferative rate (**Figure 4f**). Additional IHC staining revealed positivity for Bcl-6, weak nuclear positivity for c-MYC in approximately 30% of tumor cells, and partial positivity for Bcl-2. The following markers were negative: CD10, CD21, CD23, MUM-1, cyclin D1, and pan-cytokeratin (AE1/AE3). The overall IHC profile was consistent with DLBCL, GCB subtype.

## Staging and treatment

The patient was referred to the Department of Hematology for further evaluation. Systemic staging with 18F-fluorodeoxyglucose positron emission tomography/computed tomography (FDG-PET/CT) revealed no evidence of lymph node involvement or distant metastasis, establishing a final diagnosis of primary breast DLBCL, Ann Arbor stage IE, asymptomatic (stage IEA) with an International Prognostic Index (IPI) score of 0. The patient was initiated on the R-CHOP regimen (rituximab, cyclophosphamide, doxorubicin, vincristine, and prednisone). After completing three cycles of treatment, the patient is currently continuing the same regimen with no evidence of disease recurrence.

## Discussion

PBL is defined as lymphoma arising in the breast parenchyma with or without regional lymph node involvement, in the absence of prior lymphoma history or concomitant systemic disease. DLBCL is the predominant type, and the GCB subtype—as identified in this case—generally carries a more favorable prognosis compared to the non-GCB (activated B-cell) subtype (5, 6).

Clinically, PBL most commonly presents as a painless, unilateral breast mass in middle-aged women, without associated skin changes or nipple discharge. Imaging features are nonspecific; mammography may reveal well- or indistinct masses, and sonography typically demonstrates hypoechoic lesions with variable vascularity. According to the BI-RADS v2025 descriptors, lymphomas typically present as lobulated masses with indistinct or obscured margins and intermediate elasticity, reflecting their non-desmoplastic growth pattern, whereas invasive breast carcinomas more commonly demonstrate irregular shapes, infiltrative or spiculated margins, and marked stiffness (7). In the present case, BI-RADS 4A classification, prominent vascularity on CDFI, and intermediate-to-hard tissue stiffness on elastography raised the suspicion for malignancy, yet could not reliably distinguish PBL from primary breast carcinoma (8). Importantly, negative cytokeratin (AE1/AE3) staining in this case effectively excluded an epithelial origin, which is critical given the overlap in imaging appearances.

Although ultrasound-guided core needle biopsy (CNB) remains the standard initial diagnostic approach even for breast lesions highly suspicious for primary carcinoma, both lesions in this case were excised under the presumptive diagnosis of breast carcinoma, as preoperative clinical and imaging findings were indistinguishable from malignancy. Intraoperative frozen section raised suspicion of lymphoma, prompting immediate cessation of further resection. This highlights a critical management pitfall: PBL-DLBCL frequently mimics breast carcinoma, leading to unnecessary surgical intervention. Preoperative CNB is particularly valuable when hematologic lesions are suspected, as it avoids surgery and expedites systemic therapy (R-CHOP) (9, 10).

The diagnosis of PBL requires comprehensive IHC analysis. The expression of CD20, CD79a, and PAX-5, in concert with negativity for epithelial markers and T-cell antigens, confirms B-cell lineage. GCB subtype classification per the Hans algorithm is established by CD10 positivity or Bcl-6 positivity with MUM-1 negativity. In the present case, Bcl-6 positivity combined with MUM-1 negativity supported GCB classification despite CD10 negativity. The clear-cell-like morphology observed in this case (**Figure 3**) is atypical for conventional DLBCL and likely represents a rare morphologic variant. **Figure 3** (×400) is the highest-magnification image available; additional representative fields could not be obtained, which is acknowledged as a limitation. The Ki-67 index was high (~80%). c-MYC expression was weak and present in approximately 30% of tumor cells, which is below the ≥40% threshold for MYC overexpression. Bcl-2 showed partial positivity; however, quantitative percentage was not provided in the pathology report, precluding assessment against the ≥50% BCL2 threshold. Fluorescence *in situ* hybridization for MYC, BCL2, and BCL6 rearrangements was not performed. Consequently, the IHC profile does not meet standard criteria for double-hit suspicion, supporting the favorable prognostic profile of this case. The lack of quantitative Bcl-2 data and FISH testing is acknowledged as a limitation (3, 11).

The dimensional discrepancy between ultrasound and gross pathology measurements (10 o’clock: 19 × 12 × 14 mm vs 3.5 × 2.5 × 2.0 cm; 9 o’clock: 42 × 19 mm vs 4.5 × 3.5 × 2.5 cm) likely reflects tissue fixation artifacts, inclusion of surrounding normal tissue in the gross specimen, and differing measurement planes. Both modalities confirmed two distinct lesions, and the pathological staging was not affected.

Accurate staging by FDG-PET/CT is indispensable for treatment planning (12). R-CHOP chemoimmunotherapy remains the standard of care for DLBCL, with consolidative radiotherapy considered in selected cases of localized disease (13). The favorable prognostic profile of this patient (stage IEA, IPI 0, GCB subtype) predicts a high rate of complete remission with standard therapy (14, 15).

## Conclusion

This case underscores that PBL-DLBCL should be included in the differential diagnosis of breast masses presenting with BI-RADS 4A features, particularly when CDFI reveals prominent vascularity. Definitive diagnosis mandates thorough histopathological and IHC workup, as imaging alone is insufficient to distinguish PBL from breast carcinoma. Prompt referral to hematology, comprehensive staging, and initiation of R-CHOP therapy are essential to achieve optimal outcomes in this rare but treatable malignancy.

## Data Availability

The original contributions presented in the study are included in the article/supplementary material. Further inquiries can be directed to the corresponding author.
